# Heme Oxygenase-1 and Prostate Cancer: Function, Regulation, and Implication in Cancer Therapy

**DOI:** 10.3390/ijms25179195

**Published:** 2024-08-24

**Authors:** Ramia J. Salloom, Iman M. Ahmad, Dania Z. Sahtout, Michael J. Baine, Maher Y. Abdalla

**Affiliations:** 1 Department of Pathology, Microbiology, and Immunology, University of Nebraska Medical Center, Omaha, NE 68198, USA; ramia.salloom@unmc.edu (R.J.S.); dsahtout@unmc.edu (D.Z.S.); 2 Department of Clinical, Diagnostic, and Therapeutic Sciences, University of Nebraska Medical Center, Omaha, NE 68198, USA; iman.ahmad@unmc.edu; 3 Department of Radiation Oncology, University of Nebraska Medical Center, Omaha, NE 68198, USA; mbaine@unmc.edu

**Keywords:** HO-1, prostate cancer (PC), hypoxia, oxidative stress, metastasis, therapy resistance, immunomodulation, therapeutic strategy

## Abstract

Prostate cancer (PC) is a significant cause of mortality in men worldwide, hence the need for a comprehensive understanding of the molecular mechanisms underlying its progression and resistance to treatment. Heme oxygenase-1 (HO-1), an inducible enzyme involved in heme catabolism, has emerged as a critical player in cancer biology, including PC. This review explores the multifaceted role of HO-1 in PC, encompassing its function, regulation, and implications in cancer therapy. HO-1 influences cell proliferation, anti-apoptotic pathways, angiogenesis, and the tumor microenvironment, thereby influencing tumor growth and metastasis. HO-1 has also been associated with therapy resistance, affecting response to standard treatments. Moreover, HO-1 plays a significant role in immune modulation, affecting the tumor immune microenvironment and potentially influencing therapy outcomes. Understanding the intricate balance of HO-1 in PC is vital for developing effective therapeutic strategies. This review further explores the potential of targeting HO-1 as a therapeutic approach, highlighting challenges and opportunities. Additionally, clinical implications are discussed, focusing on the prognostic value of HO-1 expression and the development of novel combined therapies to augment PC sensitivity to standard treatment strategies. Ultimately, unraveling the complexities of HO-1 in PC biology will provide critical insights into personalized treatment approaches for PC patients.

## 1. Introduction to PC

PC is the leading cause of cancer-related deaths among men worldwide [1]. The incidence of PC varies across populations, with higher rates reported in North America, Europe, and Australia, while lower rates are observed in Asian countries [2,3]. PC poses significant challenges due to its heterogeneity and variable clinical outcomes despite advancements in screening techniques and treatment options [4].

The prostate gland is a small, walnut-shaped organ located just below the bladder and in front of the rectum in males. The primary function of the prostate is to provide nutrients to sperm [5]. Oxidative stress and inflammation within the prostate microenvironment may lead to increased cell growth, known as benign prostate hyperplasia (BPH), or genetic and epigenetic alterations in prostate epithelial cells that can generate carcinomas [6].

Several risk factors have been identified to influence PC development, including age, ethnicity, family history, and genetics [2,7]. The likelihood of developing PC increases with age, with most cases being diagnosed in men over 65 [7,8]. Ethnicity also plays a role, with higher incidence rates observed in certain populations [9]. Family history and genetic mutations further elevate the risk [9]. Additional modifiable factors can include diet, physical activity, smoking, and specific medications [10].

**Figure 1:**
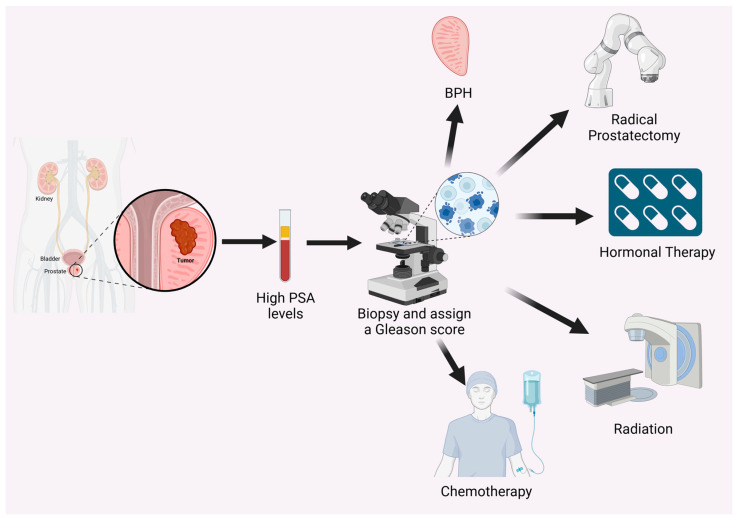
Diagnosis of PC. The diagnosis begins with the measurement of PSA levels in the blood, where elevated levels may indicate the presence of PC. If elevated PSA levels are detected, a biopsy is performed on prostate tissues, and a Gleason score is assigned to determine the aggressiveness of the disease. The biopsy results can differentiate between BPH and cancer. Tumor stage, Gleason score, and PSA levels determine the treatment strategy, which may include radical prostatectomy, hormonal therapy, radiotherapy, or chemotherapy. (Created with BioRender.com).

Prostate-specific antigen (PSA) is an enzyme that is primarily found in semen, where its primary function is to liquify semen, facilitating sperm mobility [5]. Although predominantly present in semen, a small amount of PSA circulates in the blood. The measurement of PSA levels in the blood is commonly used as a screening tool for PC [11] (Figure 1). Elevated PSA levels can indicate the presence of PC, although high levels can also result from other prostate conditions, such as BPH or prostatitis, which is an inflammation of the prostate [11,12]. PSA screening has been crucial for early detection, leading to an increase in the diagnosis of localized disease [13].

The Gleason score, which was first described by Gleason [14], is a critical tool used in evaluating the prognosis of PC. It grades cancer based on its microscopic appearance and is a strong predictor of disease aggressiveness [15] (Figure 1). Specifically, the Gleason score is the combination of the cellular grade of the most common and second most common histologic appearances within the malignancy. Cellular grade in PC ranges from 3 to 5 with Gleason score thus ranging from 6–10; with higher scores indicating more aggressive cancer and a poorer prognosis [16]. This scoring system helps guide treatment decisions and provides insights into the potential progression of the disease.

Current treatment strategies for PC are primarily determined by the patient’s risk group, which is assessed based on tumor stage, Gleason score, and PSA levels [17]. For localized disease, treatment options include radical prostatectomy and radiation therapy [17,18]. Androgen deprivation therapy (ADT), targeting the androgen receptor (AR) signaling pathway, is the mainstay of treatment for advanced PC. While initially effective, most patients eventually develop resistance to ADT, leading to the development of castration-resistant PC (CRPC) [19]. Therapeutic approaches for CRPC include novel AR-targeted agents, chemotherapy, immunotherapy, and radiopharmaceuticals [20] (Figure 1).

Despite the high 5-year survival rate of early-diagnosed PC, 97%, there remains a critical need to develop more effective therapeutic strategies due to several factors [1]. Firstly, advanced and metastatic PC has a significantly worse prognosis compared to early-stage disease, 32%, with current therapies often proving less effective [21]. Additionally, many patients with advanced PC develop resistance to standard treatments, including ADT, chemotherapy, and radiation, necessitating new approaches to overcome this resistance. Furthermore, existing treatments can have significant side effects that impact patients’ quality of life, highlighting the need for therapies that are both more effective and less damaging [22]. Moreover, even after successful initial treatment, there is a risk of recurrence, which underscores the importance of developing strategies to prevent relapse [23,24]. Finally, enhancing early detection methods to better distinguish between aggressive and indolent forms of PC can help ensure prompt and appropriate treatment [25,26]. These advancements are crucial for improving long-term outcomes and the overall well-being of patients.

To address these challenges, it is imperative to unravel the complex molecular and cellular pathways involved in PC development and progression. This review aims to shed light on a critical molecular effector, HO-1, a key enzyme in the heme degradation pathway that has emerged as a driver in PC progression and therapy resistance. We will elucidate the intricate interplay of genetic and epigenetic alterations, signaling pathways, tumor microenvironment, and therapy-induced changes that shape PC biology. By examining the existing literature and recent advancements, we hope to contribute to a deeper understanding of the molecular complexity of PC and identify potential avenues for developing more effective treatment strategies.

## 2. Introduction to HO-1

**Figure 2:**
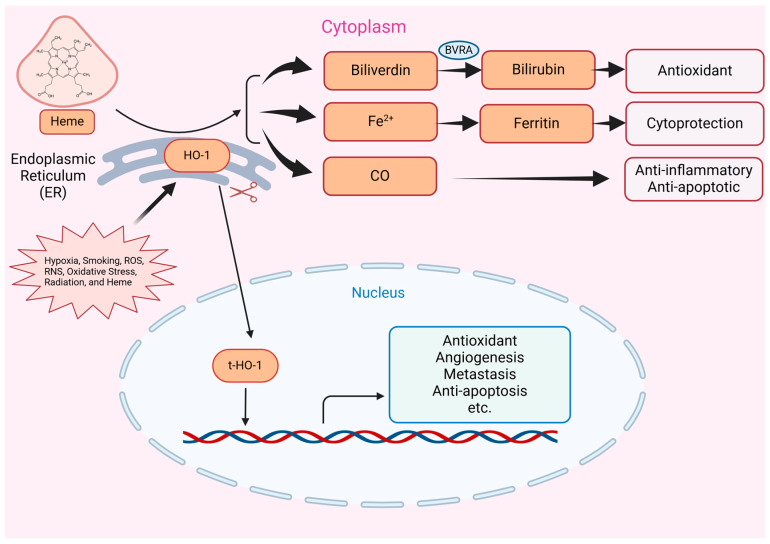
Cellular Localization and Function of HO-1. HO-1 is upregulated by various stimuli, including hypoxia, ROS, oxidative stress, and heme. HO-1 is initially synthesized and localized to the endoplasmic reticulum (ER), where it catalyzes the degradation of heme. This process produces biliverdin, which is subsequently converted to bilirubin by biliverdin reductase A (BVRA), carbon monoxide (CO), and ferrous iron (Fe^2+^). Ferrous iron is quickly sequestered into ferritin for storage. Both biliverdin and bilirubin serve as potent antioxidants, while CO exerts cytoprotective effects by activating anti-inflammatory and anti-apoptotic pathways. Under stress conditions, HO-1 undergoes proteolytic cleavage (t-HO-1) and translocates to the nucleus, where it regulates the expression of genes related to antioxidants, angiogenesis, metastasis, anti-apoptosis, and other crucial cellular processes. (Created with BioRender.com).

HO-1 stands as a pivotal and dynamic player in cellular stress response and therapy resistance, captivating the attention of researchers across various disciplines [27]. HO-1 is a member of the heme oxygenase family and is primarily localized in the endoplasmic reticulum, mitochondria, and plasma membrane [27,28] (Figure 2). Unlike its constitutively expressed counterpart, heme oxygenase-2 (HO-2), HO-1 is an inducible enzyme. Additionally, a third isoform, HO-3, has been identified in rats; however, it lacks catalytic activity [29]. Various cells within the prostate tumor microenvironment and immune cells express HO-1, such as macrophages, T cells, and dendritic cells [30,31,32]. HO-1 is induced in response to various stressors, including oxidative stress, inflammation, heavy metals, certain cytokines, and hypoxia [33]. These stimuli trigger the activation of specific signaling pathways and transcription factors, leading to the upregulation of HO-1 expression as a protective measure to combat cellular stress.

Central to the function of HO-1 is its role in the heme degradation pathway, which is critical for cellular homeostasis (Figure 2). Heme, an iron-protoporphyrin IX complex, is an essential component in various biological processes, including oxygen storage and transfer via myoglobin and hemoglobin, electron transfer through cytochromes, and signal transduction via heme-regulated inhibitors [34,35]. However, free heme is highly toxic and can contribute to the generation of harmful ROS through the Fenton reaction, necessitating its breakdown by heme oxygenases [34,35]. HO-1 enzymatically degrades heme, producing equimolar amounts of carbon monoxide (CO), biliverdin, and free iron (Fe^2+^) (Figure 2).

CO, a well-known gaseous signaling molecule, demonstrates various effects in cellular physiology, including vasodilation, anti-inflammatory actions, and modulation of apoptosis and cell proliferation [36]. Biliverdin, in turn, is rapidly converted into bilirubin, a potent antioxidant molecule known for its protective role against oxidative damage [37]. Finally, the release of free iron presents both challenges and opportunities. In contrast to bilirubin, free iron can fuel the Fenton reaction, leading to harmful reactive oxygen species (ROS) generation; it also plays a crucial role in essential cellular processes [38]. The dynamic interplay of these bioactive molecules governs diverse cellular responses to stress (Figure 2). Different studies show that CO can trigger upregulation of HO-1 by upregulating Nrf2-dependent HO-1 expression via PERK activation [39]. Furthermore, studies indicate that HO exhibits a higher affinity for binding O2 compared to other heme proteins, enabling it to effectively differentiate between CO and O2 [40]. It has been shown that the active site of HO is specifically adapted to strongly favor O2 binding over CO [40,41,42].

Emerging evidence suggests that HO-1 can translocate to the nucleus under certain stress conditions, where it may regulate gene expression [43,44] (Figure 2). Normally, HO-1 is anchored to the lumen of the smooth endoplasmic reticulum (sER) by its C-terminus. However, under stress, proteolytic cleavage leads to the truncation of the C-terminus, releasing a significant HO-1 fragment containing the N-terminus into the cytosol [45]. This fragment can migrate to the nucleus in response to specific signals, with the “nuclear shuttling sequence (NSS)” playing a critical role in facilitating HO-1’s nuclear import [45]. Once in the nucleus, HO-1 interacts with nuclear proteins that regulate transcription factors such as AP1, STAT1, STAT3, among others [46,47]. Additionally, HO-1 functions as a DNA-binding protein at specific DNA binding motifs, with three potential DNA-binding domains identified within its structure [48]. Through its interaction with DNA, HO-1 may regulate the expression of various pro-tumorigenic genes, promoting cancer cell survival, proliferation, and metastasis, independent of its enzymatic activity (Figure 2).

Within the nucleus, HO-1 interact directly with components of the DNA damage response (DDR) pathway [49,50]. Given the critical role of the DDR in maintaining genomic stability, HO-1’s involvement suggests it may help cancer cells withstand genotoxic stress, potentially leading to resistance against treatments like chemotherapy and radiation, which rely on inducing DNA damage to kill cancer cells. Additionally, studies suggest that HO-1 might interact with or influence the stability of G-quadruplexes, which are non-canonical nucleic acid structures formed in guanine-rich regions of DNA and RNA [50]. These structures play key roles in the regulation of gene expression, DNA replication, and genome stability. By stabilizing or destabilizing G-quadruplexes, nuclear HO-1 might modulate the transcription of genes involved in cell proliferation and survival, thereby influencing cancer growth. This nuclear activity of HO-1 adds another layer to its role in cancer biology, suggesting that its function extends beyond cryoprotection and into the modulation of tumor-promoting pathways. The overexpression of the nuclear HO-1 has been shown to correlate with PC aggressiveness and metastasis [32].

The induction of HO-1 reflects a cellular strategy aimed at diminishing oxidative stress and inflammation, which are pivotal factors in numerous pathological conditions, including cancer. Moreover, the broad functions of HO-1 extend beyond cellular protection, encompassing immunomodulation, tissue repair, and angiogenesis, further emphasizing the significance of this enzyme in PC development.

## 3. Regulation of HO-1 in PC

**Figure 3:**
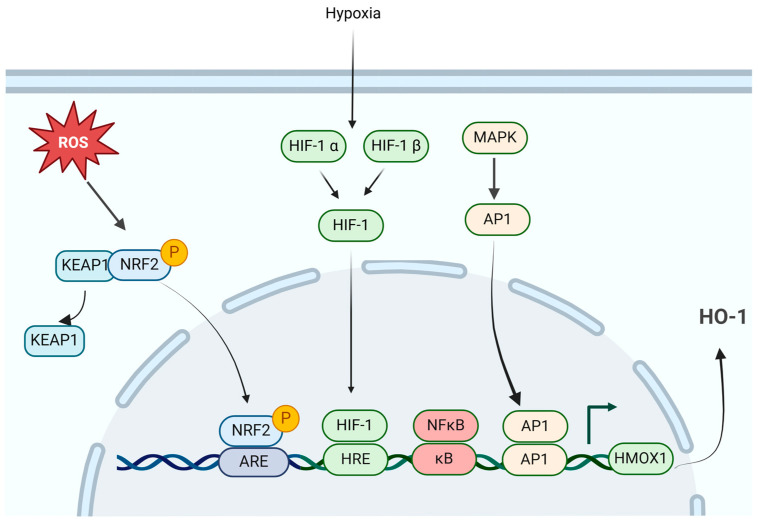
The regulation of HO-1 in PC cells. Multiple pathways regulate HO-1 expression in PC cells. The NRF2 pathway is crucial, where under oxidative stress, NRF2 dissociates from KEAP1, translocates to the nucleus, binds to ARE in the HO-1 promotor region, and activates transcription. The HIF-1α pathway also activates HO-1 transcription, under hypoxic conditions, HIF-1α translocates to the nucleus where it dimerizes with HIF-1β to form the HIF-1 complex, which binds to HREs in the HO-1 promotor. Additionally, inflammation activates NF-ƙB pathway, which translocates to the nucleus and binds the HO-1 promotor, inducing transcription. The MAPK pathway promote HO-1 expression through intermediate signaling pathways, including the activation of AP-1 transcription factor, which binds to the HO-1 promotor, inducing transcription. (Created with BioRender.com).

HO-1 has been shown to be induced in multiple cancers, including PC [51,52,53,54,55], and its elevated expression is closely associated with poor patient prognosis [44]. Regulating HO-1 expression is essential for maintaining cellular homeostasis, and dysregulation of this process has been implicated in various diseases, including cancer. In PC, the expression of HO-1 is intricately regulated by numerous signaling pathways, transcription factors, and external stimuli that impact HO-1 induction in PC cells (Figure 3).

One significant pathway involved in HO-1 induction is the nuclear factor erythroid 2-related factor 2 (NRF2) pathway, which plays the main role in inducing HO-1 expression in response to oxidative stress [56,57] (Figure 3). Under normal conditions, NRF2 is sequestered in the cytoplasm through its association with Kelch-like ECH-associated protein 1 (KEAP1). Upon exposure to oxidative stress, NRF2 dissociates from KEAP1, translocates to the nucleus, and binds to antioxidant response elements (AREs) within the HO-1 promoter region, leading to its transcriptional activation [56,58] (Figure 3). However, an alternative “hinge and latch” model has been proposed, wherein NRF2 is not merely released from KEAP1 but rather, its nuclear translocation requires de novo synthesis of NRF2 [59,60]. This model posits that even under oxidative stress, some KEAP1-bound NRF2 is degraded, and the newly synthesized NRF2 is what accumulates in the nucleus to drive the expression of target genes [59,60]. Additionally, BTB and CNC homology 1 (BACH1), a heme sensor, acts as a major transcription repressor of HO-1 by competing with NRF2 for ARE binding under normal conditions. Elevated heme levels cause BACH1 to dissociate from AREs, permitting HO-1 expression [57,61,62].

Another pathway involved in HO-1 induction is hypoxia-inducible factor 1 α (HIF-1α) pathway [63]. Under hypoxic conditions, HIF-1α escapes proteasomal degradation, translocates to the nucleus, and dimerizes with HIF-1β, forming the HIF-1 complex, which binds to hypoxia-response elements (HREs) within the HO-1 promoter to initiate transcriptional upregulation [64,65] (Figure 3). Hypoxia is a common feature of solid tumors, including PC, resulting in HIF-1-mediated induction of HO-1, contributing to tumor adaptation and angiogenesis [63,66].

In addition to these pathways, the regulation of HO-1 expression is also influenced by non-coding RNAs, including microRNAs (miRNAs) and long non-coding RNAs (lncRNAs) [67]. Recent studies have demonstrated that miRNAs can modulate HO-1 expression in specific cell types. For instance, miR-377 and miR-217 have been shown to decrease HO-1 levels in endothelial cells by directly targeting the 3′ untranslated region (UTR) of HO-1 mRNA [68]. Similarly, miR-378 has been shown to suppress HO-1 levels in lung cancer cells [69]. lncRNAs also contribute to cancer progression by regulating gene expression [70]. They can act as molecular sponges for miRNAs or directly interact with transcription factors and chromatin-modifying complexes to influence HO-1 transcription [67]. These non-coding RNAs play crucial roles in the progression of PC [71,72], though their specific impact on HO-1 expression in PC requires further investigation. This adds an additional layer of complexity to the regulation of HO-1, emphasizing their potential role in the progression and therapeutic resistance of PC.

Inflammation, a hallmark of cancer, also promotes HO-1 expression via the nuclear factor-kappa B (NF-ƙB) pathway, activated by various cytokines, growth factors, and inflammatory stimuli [73,74]. The active NF-ƙB complex translocates to the nucleus and binds directly to the HO-1 promoter, activating its transcription (Figure 3). Another pro-inflammatory transcription factor, activator protein 1 (AP1), has an identified binding site within the HMOX1 promoter, and HO-1 upregulation has been correlated with both NF- ƙB and AP1 expression [75,76] (Figure 3). Chronic inflammation in the prostate microenvironment can create a pro-survival niche for tumor cells and inducing HO-1 may contribute to this process.

While several direct transcriptional regulators control HO-1 expression, many other inducers activate transcription via intermediate signaling pathways. Key protein kinase pathways associated with cellular stress, such as the mitogen-activated protein kinases (MAPKs), including ERK, JNK, and notably p38, are involved in regulating HO-1 [75,77] (Figure 3). Additionally, the phosphatidylinositol 3-kinase (PI3K)–AKT pathway influences HO-1 expression in response to cytokines and prostaglandins, with IL-10 upregulation mediated via PI3K and STAT3 [78]. AMP-activated protein kinase (AMPK) also modulates HO-1 by interacting with its downstream effectors and NRF2 [79]. The exact process through which the protein kinase signaling pathway triggers HO-1 expression is a complex interplay involving NRF2, AP-1, and HIF-1 transcription factors [57]. Furthermore, our lab has identified STAT1 as a potential regulator of HO-1 expression. Inhibiting STAT1 with fludarabine, a specific STAT1 inhibitor, has been shown to reduce HO-1 expression in various PC cell lines [80].

ROS and oxidative stress are common occurrences in cancer cells. As an antioxidant enzyme, HO-1 is upregulated in response to increased oxidative stress to help counteract ROS-induced cellular damage and support cancer cell survival and proliferation [33]. This upregulation of HO-1 is not typically a direct induction but occurs through various signaling pathways, such as NRF2 and NF-ƙB signaling pathways [56,81]. Through these pathways, HO-1 is upregulated in a coordinated response to oxidative stress, enabling the cell to mitigate ROS-induced damage and enhance survival and proliferation in the context of cancer.

Understanding the regulation of HO-1 expression in PC is crucial for unraveling its role in disease pathogenesis and therapy response. Targeting the pathways and factors involved in HO-1 induction may hold promise as a therapeutic strategy for PC treatment and warrants further investigation. Moreover, elucidating the interplay between inflammation, oxidative stress, hypoxia, and cancer-related factors in HO-1 regulation may provide valuable insights into the mechanisms underlying disease progression and therapeutic resistance in PC. 

## 4. Role of HO-1 in PC Progression: Hypoxia

Understanding the fundamental biological events in prostate carcinogenesis is crucial to reducing PC mortality. Hypoxia, or low oxygen levels, is an early and significant event in prostate carcinogenesis, linked to aggressive cancer phenotype [33,82]. It plays a significant role in PC growth, metastasis, and the progression to hormone-refractory stages [82,83]. Hypoxia drives genetic, metabolic, and proteomic changes that enhance glycolysis, angiogenesis, stem cell-like properties, and the selection of resistant cancer clones [84,85]. Consequently, tumor hypoxia and related biomarkers are associated with poor prognosis, treatment failure, and disease relapse [85]. Targeting hypoxia-induced signaling pathways and their biological effects could offer effective strategies for preventing and treating PC.

Several signaling pathways are activated in the hypoxic microenvironment of PC, leading to the induction of HO-1. This enzyme plays a crucial role in helping cells adapt to hypoxic stress and mitigating the activation of proinflammatory pathways [86]. HO-1 overexpression is linked to improved cell survival under hypoxic conditions, highlighting its protective function and its promising therapeutic significance for PC treatment [87].

**Figure 4:**
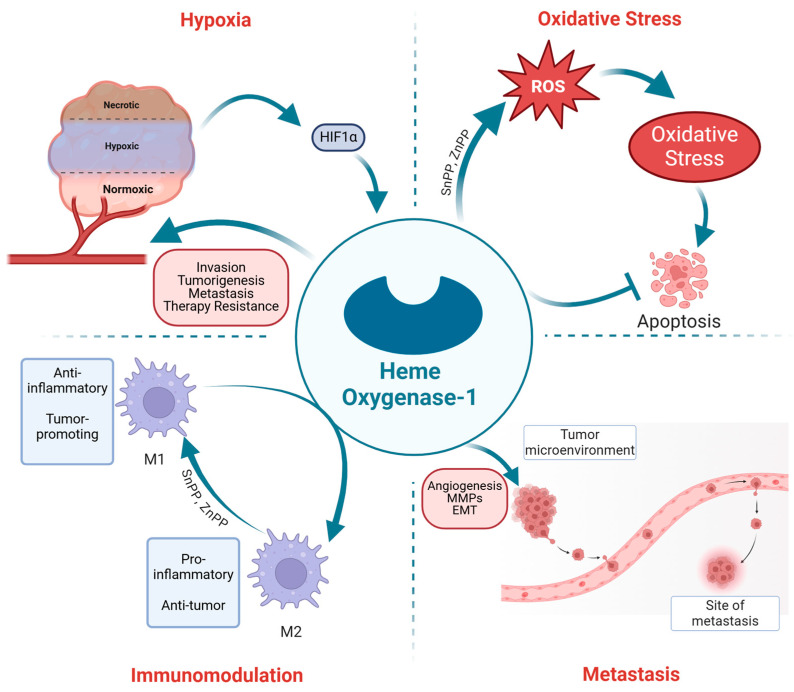
The Role of HO-1 in PC Progression. In hypoxic tumors, HIF-1α is activated, which in turn upregulates HO-1 expression. This upregulation triggers pathways that promote PC invasion, tumorigenesis, metastasis, and therapy resistance. HO-1 plays a key role in mitigating the effects of elevated ROS levels in PC cells by activating survival pathways and inhibiting apoptosis. Inhibition of HO-1 using SnPP or ZnPP results in increased ROS levels, leading to cancer cell apoptosis. Additionally, HO-1 facilitates PC metastasis by promoting angiogenesis, enhancing MMP activity, and driving EMT processes. HO-1 also exerts immunomodulatory effects, where elevated HO-1 levels are associated with the anti-inflammatory and tumor-promoting M2 phenotype. Conversely, inhibiting HO-1 activity shifts macrophage polarization towards the M1 phenotype, which is pro-inflammatory and anti-tumorigenic (arrows indicate enhancement or inhibition). (Created with BioRender.com).

Under hypoxic conditions, the HIF-1α pathway is activated, leading to upregulating in HO-1 expression [88,89]. In response to this stress, HO-1 undergoes proteolytic cleavage, resulting in its nuclear translocation, where it modulates key transcription factors, facilitating the expression of critical genes that promote cell proliferation and invasion, enhancing tumorigenesis, and activating survival genes [45,90] (Figure 4). The induction of HO-1 under hypoxia helps cancer cells to survive and adapt to the low-oxygen environment by enhancing their antioxidative capacity and promoting angiogenesis.

There is a bidirectional relationship between HO-1 and HIF-1α. While HIF-1α directly upregulates HO-1 expression under hypoxic conditions, HO-1 can also stabilize HIF-1α, creating a feedback loop that amplifies the hypoxic response. HO-1’s byproducts, particularly CO, can inhibit prolyl hydroxylases (PHDs), the enzymes responsible for HIF-1α degradation under normoxic conditions [91,92,93]. By inhibiting PHDs, HO-1 prevents the degradation of HIF-1α, leading to its accumulation and sustained activity, which further promotes the transcription of hypoxia-responsive genes.

The role of HO-1 in the hypoxic response has significant implications for therapy resistance in PC (Figure 4). Data from our lab and others has demonstrated that hypoxia-induced HO-1 upregulation contributes to the resistance of cancer cells to conventional therapies, such as radiotherapy and chemotherapy, which often rely on oxygen-dependent mechanisms to induce cell death [80,93,94]. By enhancing the antioxidant defenses of cancer cells and promoting survival pathways, HO-1 helps to protect PC cells from the cytotoxic effects of these treatments, thereby contributing to therapy resistance and disease progression.

Hypoxic regions are commonly found in various cancers, including PC, rendering these tumors resistant to therapies. Hypoxia induces a range of responses in solid tumors, such as gene expression alteration, apoptosis suppression, EMT stimulation, and enhanced angiogenesis [95,96]. Moreover, Hypoxia also enhances clonal selection leading to increased invasion and metastasis, which poses a significant challenge to conventional therapies and contributes to cancer cell resistance against ADT, radiotherapy, and chemotherapy [95,97,98] (Figure 4).

In addition, hypoxic cancer stem cells (CSC) are characterized with high expression levels of Adenosine-triphosphate binding cassette transporters (ABC transporters) [99]. These transporters constitute a highly efficient efflux system that confers chemoresistance in cancer cells [100]. Activation of these ABC transporters by HIFs upon hypoxia conditions diminishes the effect of chemotherapy agents, thereby exacerbating chemoresistance [99].

In conclusion, the upregulation of HO-1 in response to hypoxia plays a crucial role in PC progression by enhancing cell survival, promoting angiogenesis, and contributing to therapy resistance. Therefore, employing HO-1 inhibitors alongside standard therapies could overcome resistance and improve treatment outcomes for PC patients.

## 5. The Interplay of HO-1, Oxidative Stress, and PC

Carcinogenesis is a complex process involving genetic alterations and dysregulated cell proliferation [65]. Oxidative stress, characterized by an imbalance between ROS production and antioxidant defense mechanisms, has been implicated in cancer initiation and progression [101,102]. Chronic oxidative stress leads to DNA damage, inflammation, and cellular dysfunction, contributing to carcinogenesis [103,104,105]. Enzymatic antioxidants such as superoxide dismutase (SOD), catalase, and glutathione peroxidase (GPx), along with non-enzymatic antioxidants like glutathione (GSH), play crucial roles in neutralizing ROS and maintaining cellular redox homeostasis [106,107,108,109].

HO-1 is a stress-inducible enzyme activated by various stimuli including oxidants, cytokines, and heavy metals [110]. While HO-1 exhibits cytoprotective effects in normal cells by mitigating oxidative stress and inflammation [111,112], its overexpression is observed in several cancer types, including PC, where it contributes to tumor cell survival and resistance to therapy [33,55,113,114]. Understanding the mechanisms underlying HO-1-mediated redox regulation in cancer cells is critical for developing effective therapeutic strategies. Combinatorial approaches targeting HO-1 alongside conventional chemotherapy or radiotherapy hold promise for overcoming treatment resistance in PC [113].

HO -1 serves as the rate-controlling enzyme in cellular heme catabolism, catalyzing the breakdown of heme moieties into equimolar amounts of CO, Fe, and biliverdin, which is subsequently converted to bilirubin by biliverdin reductase [115,116] (Figure 2). HO-1’s cellular protective effects stem from its ability to suppress inflammation and oxidative stress [117]. Bilirubin, a byproduct of heme degradation, exhibits antioxidant properties and likely contributes to the mammalian body’s redox balance by scavenging ROS [118,119,120]. Moreover, CO exerts physiological functions, including anti-inflammatory and anti-apoptotic effects mediated through the mitogen-activated protein kinase (MAPK) pathway, as well as vasodilatory effects [121,122]. Free iron generated by HO-1 activity is sequestered by ferritin, thereby preventing hydroxyl radical formation via the Fenton reaction [123].

Notably, numerous studies, including investigations conducted in our laboratory, have elucidated the multifaceted roles of HO-1 in cellular processes such as growth regulation, angiogenesis, response to bacterial infection, and modulation of macrophage polarization towards anti-inflammatory (M2) or pro-inflammatory (M1) phenotypes [124,125,126]. The antioxidant properties of HO-1 enable cancer cells to evade ROS-induced DNA damage and apoptosis (Figure 4), thereby conferring resistance to chemotherapy and radiotherapy [113,114]. Consequently, the combined administration of chemotherapy and/or radiation therapy with HO-1-inhibiting agents holds promise for overcoming therapeutic resistance in cancer treatment.

Clinical studies have reported that HO-1 expression is increased in PC patients, which correlates with increased serum PSA, resistance to therapy, and poor clinical outcomes [127,128,129,130]. It has been shown that overexpression of HO-1 correlates with PC growth, aggressiveness, and metastasis [50,127,131]. However, conflicting opinions have emerged on whether HO-1 provides growth advantages and metastasis to PC cells or promotes cell death [132]. Several concerns should be addressed. The available literature suggests that the effects of HO-1 depend on the amount of ROS. Low ROS levels enhance cell survival, while high levels promote cell death [133,134] (Figure 4). Under oxidative stress, increased ROS levels enhance HO-1 expression, thereby protecting PC cells by mitigating the deleterious effects of ROS, resulting in metastasis and resistance to cancer treatments [135]. However, HO-1 inhibition in PC tilts the balance towards ROS overproduction, promoting cell death and inhibiting PC proliferation and metastasis [136,137] (Figure 4). Thus, targeting HO-1 pathways may serve as a novel therapeutic strategy in PC therapy.

## 6. HO-1 and PC Metastasis

HO-1 plays a critical role in the metastatic progression of PC. Metastasis, the spread of cancer cells from the primary tumor to distant organs, is a significant cause of morbidity and mortality among PC patients [138,139,140]. Understanding the underlying mechanisms of metastasis in PC is a critical aspect to improving cancer prognosis. As mentioned previously, HO-1 is notably upregulated in response to various stressors, including hypoxia, which is common in the tumor microenvironment [141]. This upregulation is essential not only for the survival of cancer cells under stress but also for their metastatic capabilities [142]. 

HO-1 enzymatic byproduct, such as biliverdin and CO, possess pro-angiogenic properties [27,44,143]. These catabolic products contribute to forming new blood vessels by activating VEGF, a key regulator of angiogenesis, which is vital for supplying nutrients and oxygen to growing tumors [144,145]. By driving angiogenesis, HO-1 supports tumor expansion and enables cancer cells to enter the bloodstream and metastasize to other parts of the body [146,147,148] (Figure 4).

Furthermore, HO-1 enhances the invasive and migratory abilities of PC cells by modulating the expression of matrix metalloproteinases (MMPs), which degrade the extracellular matrix and facilitate cancer cell invasion [149,150]. Additionally, HO-1 influences the epithelial-mesenchymal transition (EMT), a process where epithelial cells acquire mesenchymal properties, increasing their motility and invasiveness [151,152] (Figure 4). Studies, including those from our lab, have demonstrated that inhibiting HO-1 can downregulate the expression of EMT markers and attenuate PC cell migration [31,80].

Furthermore, aggressive prostate tumors often induce supportive changes in adjacent benign tissues, one of which is the increased expression of HO-1 [32]. HO-1 is predominantly produced by macrophages that accumulate in tumor regions and at the tumor-invasive front [153,154]. This is evident in both rat models and human clinical samples. In rat prostate tumors, small metastatic tumors were more effective at attracting HO-1^+^ macrophages compared to larger non-metastatic ones [32]. Similarly, in human samples, high-grade tumors and bone metastases showed significant accumulation of HO-1^+^ macrophages at invasive fronts, which correlated with bone metastases presence and lower AR expression [32]. This suggests that HO-1^+^ macrophages play a crucial role in the metastatic progression of PC.

Recent studies suggest that hypoxic cancer cells, including PC cells, release more exosomes loaded with bioactive molecules such as proteins, lipids, and nucleic acids [155,156]. These exosomes can alter the tumor microenvironment and promote metastasis [157,158]. Further supporting this, studies indicate that exosomal long noncoding RNA (lncRNA) HOXD-AS1 is upregulated in CRPC and serum exosomes from metastatic PC patients [156]. Exosomal HOXD-AS1 promotes metastasis by modulating the miR-361-5p/FOXM1 axis, enhancing cancer cell invasiveness. Additionally, serum exosomal HOXD-AS1 levels correlate with higher Gleason scores and worse clinical outcomes, highlighting its potential as a biomarker for detecting and treating metastatic PC [156]. HO-1 upregulation has been linked to increased exosome production, further facilitating the metastatic process.

Additionally, the hypoxic response promotes the expression of genes involved in angiogenesis, invasion, and survival under low oxygen conditions [159]. Furthermore, the immunomodulating capabilities of HO-1 in PC, which will be described later in detail, exert anti-inflammatory effects, which can suppress anti-tumor immunity [159]. This immune modulation creates a more favorable environment for cancer cells to thrive and spread.

The role of HO-1 in PC metastasis underscores its potential as a therapeutic target. Inhibiting HO-1 could disrupt the various pathways it influences, thereby impairing the metastatic capabilities of cancer cells. This approach could be particularly beneficial when combined with conventional therapies, which may be less effective against metastatic disease due to the protective effects conferred by HO-1. 

## 7. HO-1 and Therapy Resistance in PC

HO-1 has emerged as a significant player in therapy resistance mechanisms observed in PC [27,33,159,160], encompassing resistance to ADT, radiation therapy, and chemotherapy. Resistance to ADT is a pivotal challenge in the treatment landscape of PC [161]. As the primary treatment for PC, ADT aims to suppress the AR signaling pathway, thereby inhibiting the growth and proliferation of PC cells [162]. However, despite the initial efficacy of traditional treatment options, many patients eventually develop resistance, leading to the progression of PC into CRPC. The emergence of CRPC poses significant clinical hurdles and necessitates a deeper understanding of the underlying resistance mechanisms [163,164].

While growing evidence suggests that HO-1 may be involved in resistance to ADT in PC, the specific role of HO-1 in ADT resistance is still an area of active research and ongoing investigation [33]. Several studies have reported elevated levels of HO-1 in CRPC, and its upregulation has been implicated in several resistance mechanisms [159,165,166]. One such mechanism involves the anti-inflammatory products of heme degradation that protect the cancer cell against the oxidative damage caused by therapies [142,159]. Additionally, HO-1-mediated induction of anti-apoptotic pathways and suppression of apoptotic signaling contribute to enhanced survival of PC cells under androgen-deprived conditions [143]. These observations underscore the critical need for further research to elucidate the precise molecular mechanisms through which HO-1 contributes to ADT resistance and to clarify its impact on AR expression, the principal driver of PC progression.

Following ADT, resistance to radiation therapy also presents a significant challenge in PC management. Radiation therapy is commonly employed for localized and locally advanced PC, aiming to eradicate cancer cells through targeted radiation-induced DNA damage [113,167]. However, resistance to radiation therapy often develops, leading to treatment failure and disease recurrence [168]. In the context of radiation resistance, HO-1’s role extends to multiple facets of cellular response to radiation-induced stress. HO-1 upregulation has been associated with enhanced cellular antioxidant defenses, which can mitigate the cytotoxic effects of radiation-induced oxidative stress [168]. Additionally, HO-1 may promote DNA damage repair mechanisms enabling cancer cells to survive and proliferate despite radiation exposure [169]. Furthermore, HO-1 inhibition enhanced the radiosensitivity of human non-small lung cancer [170]. 

Chemotherapy resistance further complicates PC management, particularly in the advanced stages of the disease [169]. HO-1’s multifaceted role in chemotherapy resistance encompasses mechanisms such as enhanced antioxidant defenses, modulation of apoptotic pathways, and promotion of pro-survival signaling pathways [44,159,160]. Clinical evidence highlights the possible correlation between HO-1 expression and PC growth, aggressiveness, metastasized tumor, resistance to therapy, and poor clinical outcome [159]. Furthermore, research conducted in our lab has shown that inhibiting HO-1 enhances the chemosensitivity of PC cells by modulating several interconnected pathways related to apoptosis, including increased ROS production, disruption of the glutathione cycle, and modulation of the STAT1 pathway, in addition to attenuating cell migration [80]. Understanding the complex interplay between HO-1 and therapy resistance mechanisms is crucial for developing effective treatment strategies to improve treatment outcomes in PC. 

Moreover, HO-1’s immunomodulatory properties may further exacerbate resistance to therapies. By promoting immune evasion and dampening anti-tumor immune responses, HO-1 creates a microenvironment conducive to cancer cell survival and proliferation despite oxidative stress caused by different therapies [171]. 

The role of HO-1 in therapy resistance underscores its potential as a therapeutic target to enhance treatment efficacy and overcome acquired resistance. Our lab previously showed that inhibiting HO-1 suppressed cell proliferation and increased the efficacy of chemotherapy treatment in pancreatic ductal adenocarcinoma and PC [80,126]. Therefore, strategies aimed at inhibiting HO-1 activity or disrupting its downstream signaling pathways hold promise for improving therapy outcomes and prolonging PC patient’s survival. 

## 8. Immunomodulation by HO-1 in PC

Overexpression of HO-1 in cancer cells, including PC, has been shown to promote tumor progression through multiple mechanisms, including the modulation and enforcing the immune suppressive characteristics of the TME. Both malignant tumor cells and stromal cells within the TME can express HO-1 [172,173,174]. The catabolites of heme degradation can also diffuse outside of the cell, modulating the wider TME and influencing cellular functionality and biological processes that promote tumor progression, such as facilitating angiogenesis and metastasis, as well as fostering anti-inflammatory and immune-suppressive environments [172].

Tumor-associated macrophages (TAMs) represent the most abundant stromal cell type in the TME and the primary source of HO-1 in murine models and humans [52,173]. TAMs respond to the TME and adapt a variety of phenotypes, including pro-inflammatory (M1) and anti-inflammatory (M2) phenotypes, each with distinct gene expression and cytokine profiles [175]. HO-1 upregulation has been associated with the M2 phenotype, which is the tumor-promoting macrophage phenotype, suggesting that HO-1 may contribute to macrophage polarization [176,177,178].

Other immune cells in the TME, such as dendritic cells (DC), CD4^+^ T cells, CD8^+^ T cells, and Foxp3^+^ regulatory T cells (Treg), have been shown to express HO-1, which can influence their activity and suppress their immunogenicity [30]. Since the catabolites of heme degradation diffuse to the extracellular matrix, they may also affect other immune cells that don’t express HO-1, influencing their polarization and recruitment.

Oncogenes in cancer cells or genetic mutations in the HMOX1 promotor, the gene encoding HO-1, can drive the constitutive expression of HO-1, leading to potent immunomodulating anti-tumor immune responses. The immunomodulating role of HO-1 supports its consideration as an innate immune checkpoint [52]. Blocking immune checkpoints, which are regulatory proteins that suppress T-cell activity and immune responses, has resulted in an unprecedented clinical response in cancer patients [179,180]. In parallel comparison, inhibition of HO-1 displayed superior immunological control of tumor growth in a murine mammary adenocarcinoma model compared to anti-PD1 immunotherapy [52]. This observation underscores the potential of targeting HO-1 as a novel immunotherapy approach.

Heme degradation products, such as bilirubin and CO, have significant immunomodulatory effects [30]. The upregulation of HO-1 in PC has been correlated with advanced disease stages, cancer progression, and metastasis. Specifically, HO-1-expressing macrophages have been implicated in contributing to bone metastasis [32]. Furthermore, inhibiting HO-1 has been shown to enhance the efficacy of certain cancer treatments in PC, suggesting that HO-1 inhibition may improve the overall immune response against PC and increase the effectiveness of other therapies [129]. Although the collective evidence underscores the role of HO-1 in immunomodulation within PC, the precise function of HO-1 in PC remains incompletely understood and continues to be an active area of research.

In conclusion, HO-1-induced immunomodulation may contribute to immune evasion and therapy resistance in PC. By suppressing immune-mediated anti-tumor responses, HO-1 creates an immunosuppressive microenvironment that fosters cancer cell survival and proliferation. Understanding the complex interplay between HO-1 and therapy resistance mechanisms is crucial for developing effective strategies to overcome resistance and improve treatment outcomes in PC.

## 9. Targeting HO-1 as a Therapeutic Strategy

HO-1 has emerged as a promising therapeutic target in various diseases due to its inducible nature. This inducibility allows for targeted inhibition using metalloporphyrins (MPs), compounds structurally similar to heme but with a metal ion, such as tin or zinc, replacing the iron in heme [135]. MPs bind to HO-1 with a higher affinity than heme, inhibiting its function. These elements can modulate HO-1 activity, providing potential avenues for therapeutic intervention [181]. In addition to MPs, non-porphyrin-based inhibitors are also being explored. These include small molecule inhibitors like OB-24, as well as RNA interference techniques such as small interfering RNA (siRNA) and short hairpin RNA (shRNA) [129,135,182,183,184,185]. 

The strategy of HO-1 inhibition first emerged as a promising therapeutic approach in the late 1980s [186]. Research has shown that overexpression of HO-1 is strongly correlated with elevated bilirubin levels, leading to neonatal hyperbilirubinemia [187]. Based on these findings, studies have investigated how HO-1 inhibition can reduce bilirubin levels in newborns and subsequently mitigate hyperbilirubinemia [188]. Furthermore, HO-1 inhibition has shown therapeutic potential across a broad spectrum of diseases, including M. Tuberculosis (TB) and Alzheimer’s disease [189,190,191,192]. However, the function of HO-1 in PC still needs to be fully elucidated.

As mentioned previously, HO-1 exhibits anti-inflammatory effects by reducing pro-inflammatory mediators, positioning it as an attractive target in PC, where inflammation significantly contributes to disease progression [193,194]. Hypoxia plays a significant role in PC progression and metastasis activating the expression of multiple pathways that mediate adaptation including HO-1 [82,87]. Moreover, HO-1 promotes tumor growth by inducing angiogenesis, primarily through the expression of angiogenic factors such as VEGF [175,176]. Additionally, HO-1 facilitates PC metastasis, leading to advanced disease stages and compromising the efficacy of localized treatments. HO-1 modulates the tumor microenvironment by fostering pro-inflammatory niches supporting PC survival. Importantly, HO-1 also confers resistance to ADT, radiotherapy, and chemotherapy by activating anti-apoptotic pathways, enhancing cellular antioxidant defenses, and promoting DNA repair mechanisms. 

Interestingly, both induction and inhibition of HO-1 have shown anticancer effects in PC models [55,132,141]. HO-1 effects in PC depend on whether the cells are normal or malignant and the level of HO-1 activity. Evidence suggests HO-1 has dual roles in PC, influenced by cellular iron and ROS levels [33]. Low to moderate levels of ROS reinforce HO-1’s protective role by promoting anti-apoptotic pathways, thereby facilitating PC progression and enhancing cancer cell survival, treatment resistance, and metastasis [159]. Targeting HO-1 in PC can mitigate these protective effects by elevating ROS levels in cancer cells, suppressing cellular survival, and increasing tumor cell sensitivity to therapies. These excessive ROS levels trigger programmed cell death pathways, which in turn suppress tumor growth.

Clinical studies have shown increased HO-1 expression in PC patients, correlating with higher Gleason grades, elevated PSA, therapy resistance, and poor clinical outcomes [131,195]. Consequently, regulating HO-1 expression represents a promising avenue in the landscape of PC treatment, and HO-1 could also be a valuable biomarker for PC progression. In normal cells, HO-1 induction provides cytoprotection by neutralizing oxidative stress, thereby inhibiting PC growth. Thus, HO-1 represents a potential target for therapeutic prevention by enhancing its protective functions in normal cells while also serving as a treatment strategy by inhibiting PC progression and angiogenesis.

Targeting HO-1 could revolutionize various stages of PC treatment. For newly diagnosed patients undergoing RT or radical prostatectomy, an HO-1 inhibitor might enhance the efficacy of these treatments by reducing therapy resistance, potentially leading to better patient outcomes and higher cure rates. In patients with de novo metastatic disease, inhibiting HO-1 could limit the cancer’s ability to spread further and enhance the effectiveness of systemic therapies, offering new hope in combating advanced stages of the disease. Even in patients with relapsed PC, where treatment options become limited and less effective, HO-1 inhibition could provide a new avenue to re-sensitize cancer cells to existing therapies. These multifaceted roles highlight the potential of targeting HO-1 as a therapeutic strategy in PC. 

## 10. Future Perspectives and Conclusions

Targeting HO-1 or its downstream effectors may offer promising avenues for enhancing the efficacy of existing therapies and overcoming resistance in PC treatment. The clinical application of HO-1 inhibition is still in its early stages, with ongoing research focusing on optimizing their efficacy and minimizing side effects. Future studies are needed to better understand the molecular pathways regulated by HO-1 and to identify biomarkers for selecting patients who might benefit the most from HO-1-targeted therapies. Additionally, exploring the role of HO-1 in combination with standard treatment modalities, such as hormonal therapy, radiotherapy, and chemotherapy, for more effective cancer treatments.

In conclusion, Targeting HO-1 represents a novel and promising approach in cancer therapy, including PC. By inhibiting this key enzyme, it may be possible to disrupt crucial pathways involved in tumor growth, metastasis, and immune evasion, thereby improving patient outcomes. As research progresses, the development of HO-1 inhibitors and their integration into clinical practice holds the potential to revolutionize cancer treatment strategies.
